# Association between Glycosylated Hemoglobin and Serum Uric Acid: A US NHANES 2011–2020

**DOI:** 10.1155/2024/5341646

**Published:** 2024-03-14

**Authors:** Huan Li, Mingliang Sun, Chengcheng Huang, Jingwu Wang, Yanqin Huang

**Affiliations:** ^1^First Clinical Medical College, Shandong University of Traditional Chinese Medicine, Jinan 250014, China; ^2^Department of Endocrinology, Affiliated Hospital of Shandong University of Traditional Chinese Medicine, Jinan 250014, China

## Abstract

**Background:**

Serum uric acid (SUA) and glycosylated hemoglobin (HbA1c) were closely related to the body's metabolism. This study aimed to investigate the relationship between HbA1c and SUA in adults.

**Methods:**

This study selected 7293 participants aged ≥20 from 2011 to 2020 in the National Health and Nutrition Examination Survey (NHANES). The multivariate linear regression model was used to test the association between HbA1c and SUA. Subgroup analysis was performed according to age, gender, race, and body mass index (BMI). This study solved the relationship between HbA1c and SUA by fitting a smooth curve. Finally, the inflection point in the nonlinear relationship was calculated by the recursive algorithm, and the relationship between HbA1c and SUA on both sides of the inflection point was analyzed by the two-segment piecewise linear regression model.

**Results:**

All 7293 participants found a negative correlation between HbA1c and SUA by completely adjusting the model (*β* = −7.93 and 95% CI: −9.49–−6.37). In addition, when this study was stratified by gender, age, race, and BMI status, this negative correlation was still statistically significant. In the subgroup analysis, we found that the relationship between the two had different results due to gender differences. In men, HbA1c had a significant negative correlation with SUA. However, in women, the HbA1c value was positively correlated with SUA before 6.8%, and the HbA1c value was negatively correlated with SUA after 6.8%, which indicates that the relationship between HbA1c and SUA in women has changed in prediabetes and diabetes.

**Conclusion:**

This study shows that HbA1c is positively correlated with SUA in American adults before 7%. There is a negative correlation after the HbA1c value of 7%.

## 1. Introduction

Diabetes mellitus (DM) is a chronic metabolic disease characterized by increased blood glucose caused by insulin resistance or insufficient insulin secretion. Long-term hyperglycemia could cause a series of complications, which indirectly lead to metabolic disorders. Diabetes is now a serious problem worldwide, which brings a heavy public health burden to society. Serum uric acid (SUA) was a metabolite of purine decomposition. The increase in SUA was caused mainly by excessive synthesis or imbalance of uric acid (UA) excretion in the body. The rise in SUA has been confirmed to be closely related to chronic metabolic diseases, such as obesity and T2DM. Studies have demonstrated that elevated SUA might affect the body's metabolism through insulin resistance, inflammatory response, oxidative stress, and so on [1]. A population-based cohort study showed hyperuricemia was an independent risk factor for T2DM [2].

Studies have shown that SUA was closely related to HbA1c and urinary microalbumin in diabetic patients. It was essential for diabetic patients to check HbA1c levels regularly and prevent renal damage [3]. A retrospective study in China found that SUA was positively correlated with hyperinsulinemia and insulin resistance in prediabetic patients [4]. However, a study of the Chinese population found that HbA1c was negatively correlated with SUA in diabetic patients, but HbA1c was positively correlated with hemoglobin glycosylation index in women without diabetes [5]. Studies have also shown an inverted U-shaped relationship between HbA1c and SUA in both sexes and men's inflection point reaches earlier than women's [6].

The information on the association between HbA1c and SUA must still be expanded and completed. Therefore, this study aimed to explore the relationship between HbA1c and SUA using NHANES data. At the same time, based on the previous research, we considered that HbA1c and SUA had gender, age, race, BMI, and other differences, so further subgroup analysis was conducted to illustrate the association between the two in different subgroups.

## 2. Materials and Methods

### 2.1. Study Population

The data of this cross-sectional study were from the 2011–2020 National Health and Nutrition Examination Survey (NHANES). NHANES was a representative sample health and nutritional status study conducted by the National Health Statistics Center. All NHANES procedures are reviewed and approved by the National Center for Health Statistics Research Ethics Review Board, and written informed consent was obtained from all participants in the annual survey. Our current study does not contain any human identification materials. Therefore, this study does not require further ethical review, and all data can be downloaded from NHANES's official website.

In this study, 45462 participants were enrolled in the NHANES cycle from 2011 to 2020, and our inclusion criteria were 26280 subjects over 20 years old. We excluded 1830 subjects with incomplete HbA1c and 2429 subjects with incomplete SUA data. Finally, subjects with complete HbA1c, SUA, and other covariate data were included in our subsequent analysis (*n* = 7293).

### 2.2. Evaluation of HbA1c

HbA1c could reflect the average blood glucose level over the past 2-3 months. HbA1c has been recommended as a biomarker for detecting and monitoring diabetes, especially T2DM. This indicator was measured by diluting the whole blood sample with a hemolytic solution and injecting a small portion of the processed sample into an HPLC analysis column. The separation was achieved by using the difference in ion interaction between the cation exchange group on the surface of the column resin and the hemoglobin component. A stepwise elution buffer removed the hemoglobin fraction from the column material. The separated hemoglobin components were flowed by a photometer. The changes in absorbance were measured at 415 nm and then the analyzer calculated the relative percentage of each hemoglobin component. The HbA1c results of the NGSP network in this study are expressed using the following equation with the IFCC network: NGSP (%) = 0.09148 × IFCC (mmol/mol) + 2.1522. In obtaining glycated hemoglobin values, interinstrument quality control mainly involves analyzing monthly NGSP monitoring samples on each Tosoh G8 HPLC instrument to verify the consistency between instruments. Acceptability was defined as the estimated standard deviation of repeated differences in the sample, which should not exceed 0.229, to ensure the accuracy of the numerical values. Due to the collection of glycated hemoglobin data from the past 10 years in this study, a comprehensive analysis showed that the coefficient of variation was approximately between 0.4 and 1.4. We considered HbA1c a continuous variable in this analysis and grouped participants according to HbA1c quartiles for further analysis. In this study, HbA1c was designed as an exposure variable.

### 2.3. Evaluation of SUA

SUA data were obtained from serum samples of NHANES participants obtained in MEC. The concentration of SUA was mainly determined by using the timing endpoint method. The concentration of SUA was calculated by monitoring the absorbance change of colored products produced by the reaction of hydrogen peroxide produced by UA oxidation with 4-amino antipyrine catalyzed by 3,5-dichloro-2-hydroxybenzenesulfonate. Due to the collection of nearly 10 years of USA data in this study, a comprehensive analysis showed that the coefficient of variation is approximately between 0.9 and 2.3. In this analysis, SUA was designed as an outcome variable.

### 2.4. Evaluation of Other Covariates

Other variables in this study included age (years), gender (male/female), race (Mexican American/other Hispanic/non-Hispanic white/non-Hispanic black/other race), the ratio of household income to poverty, an education level (lower than high school/high school or general education development/high school and above), body mass index (BMI, kg/m^2^, calculated as body weight (kg) divided by the square of height (m)), energy and nutrition intake in the diet, blood pressure level (systolic blood pressure and diastolic blood pressure), renal function level (urinary albumin, urinary creatinine and urinary albumin creatinine ratio), blood lipid level (low-density lipoprotein cholesterol, high-density lipoprotein cholesterol, triglyceride, and total cholesterol), smoking status (whether or not to smoke at least 100 cigarettes in a lifetime), and drinking status (once drinking 4/5 and more sitting time per day). The criteria for selecting covariates are based on previously published studies and variables [3, 7]. The data used in this study can be found on the NHANES website.

### 2.5. Statistical Analysis

**Figure 1:**
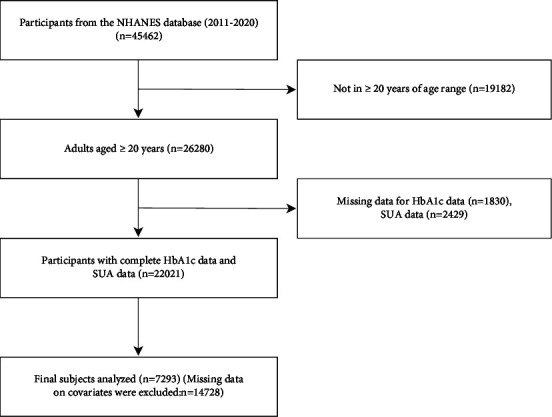
Flowchart of participant selection. NHANES, National Health and Nutrition Examination Survey; HbA1c, glycosylated hemoglobin; SUA, serum uric acid.

According to the NHANES analysis guidelines, the baseline data in this study were expressed in terms of the mean value ± standard deviation (SD) of continuous variables and the frequency (percentage) of categorical variables. This study used a multivariate linear regression model to estimate the influence of *β*s and its 95% confidence interval on the relationship between HbA1c and SUA. HbA1c was analyzed as a continuous variable and categorical variable (quartile), and the baseline characteristic difference of the HbA1c quartile was compared using the one-way analysis of variance (ANOVA) test of a continuous variable and the chi-square test of categorical variables. No covariates were adjusted in the model 1 analysis. Age, gender, and race were changed in the analysis of model 2. Analysis of model 3 based on model 2, the education level (lower than high school, high school, and high school or above), the ratio of family income to poverty, BMI, energy and nutrition intake (energy, protein, carbohydrate, dietary fiber, and fat), blood pressure level (diastolic blood pressure and systolic blood pressure), renal function level (urinary albumin, urinary creatinine, and urinary albumin creatinine ratio), blood lipid level (low-density lipoprotein cholesterol, direct high-density lipoprotein cholesterol, total cholesterol, and triglyceride), and health-related behaviors (smoking status, drinking status, and sedentary time) were also analyzed. In addition, subgroup analysis was performed by stratified multiple regression analysis by age (≥20, <40 or ≥ 40, <60 or ≥ 60, and <80 or ≥ 80 years), gender (female or male), race (Mexican American or other Hispanic or non-Hispanic white or non-Hispanic black or other race), and BMI level (<18.5 or ≥ 18.5, <25 or ≥ 25, and <30 or ≥ 30). In addition, this study solved the relationship between HbA1c and SUA by using smooth and generalized additive models. This study will use a recursive algorithm to calculate the inflection point when nonlinearity is detected. Then, a two-stage piecewise linear regression model was used to analyze the relationship between HbA1c and SUA on both sides of the inflection point. All analyses of this study were conducted using R software for statistical computing and graphics (R version 4.0.3; https://www.R-project.org) and EmpowerStats (https://www.empowerstats.com). Bilateral *P* values <0.05 were considered statistically significant. The specific process can be seen in Figure 1.

## 3. Result

### 3.1. Baseline Characteristics of Participants

A total of 7293 participants (3816 males and 3477 females) were included in this study, with an average age of 49.3 ± 17.3 years. The mean HbA1c was 5.8 ± 1.2%. The mean SUA was 328.3 ± 85.0 *μ*mol/L overall, 314.3 ± 83.9 *μ*mol/L, 316.6 ± 80.2 *μ*mol/L, 326.3 ± 79.9 *μ*mol/L, and 350.1 ± 91.0 *μ*mol/L for Quintiles 1, 2, 3, and 4. All variables in this study were significantly different from the baseline characteristics of the HbA1c quartiles. Compared with other subgroups, participants with the highest quartile of SUA were more likely to be older, male, or non-Hispanic white, with less energy, protein, carbohydrate, dietary fiber and total fat intake, lower low-density lipoprotein cholesterol, high-density lipoprotein cholesterol and total cholesterol, higher triglyceride, lower urinary creatinine, higher urinary albumin and urinary albumin ratio creatinine, higher HbA1c and blood pressure, preference for alcohol and smoking, and longer sedentary time. The baseline characteristics of all participants are shown in Table 1.

### 3.2. The Relationship between HbA1c and SUA

The results of this study indicate that higher Hb1Ac was associated with a reduced likelihood of SUA. A positive correlation between Hb1Ac and SUA was detected in the unadjusted model (*β* = 2.89, 95% CI: 1.19–4.60, and *P*=0.0009). After adjusting for confounding factors (age, gender, and race), there was no such correlation in model 2 (*β* = −0.82, 95% CI: −2.44–0.80, and *P*=0.3196), and after completely adjusting for covariates, we found that subjects with higher unit Hb1Ac had a 7.93 *μ*mol/L decrease in SUA level (model 3 *β* = −7.93 and 95% CI: −9.49–−6.37). When we converted Hb1Ac from a continuous variable to a categorical variable (quartile), we found that this association between Hb1Ac and SUA was still statistically significant at higher Hb1Ac. At the same time, subjects with the highest quartile of 1 unit Hb1Ac had an 11.79 *μ*mol/L decrease in SUA value compared with those with the lowest quartile of WWI (model 3 *β* = −11.79, 95% CI: −14.08 to −9.50).  Table 2 shows the results of multiple regression analysis of Hb1Ac and SUA.

Subgroup analyses by sex, age, race, and BMI, (reported in Table 2), showed a negative correlation between Hb1Ac and SUA still existed in men (*β* = −10.38, 95% CI = −12.51–−8.24, and *P* < 0.0001), women (*β* = −5.06, 95% CI = −7.31–−2.81, and *P* < 0.0001), age from ≥20 to <40 (*β* = −6.55 and 95% CI: −9.81–−3.29, *P* < 0.0001), age range from ≥40 to <60 (*β* = −9.44, 95% CI: −11.68–−7.20, and *P* < 0.0001), age range from ≥60 to <80 (*β* = −6.77, 95% CI: −9.84–−3.71, and *P* < 0.0001), and Mexican Americans (*β* = −10.58, 95% CI: −14.24–−6.91, and *P* < 0.0001), other Hispanics (*β* = −11.04, 95% CI: −15.61–−6.47, and *P* < 0.0001), non-Hispanic whites (*β* = −9.38, 95% CI: −12.39–−6.36, and *P* < 0.0001), non-Hispanic blacks (*β* = −5.95, 95% CI: −8.70 to −3.20, *P* < 0.0001), and other races/nationalities (*β* = −6.67, 95% CI = −11.64–−1.68, and *P*=0.0088). However, there was no correlation between the two groups of people aged ≥80 years.

Findings from this study showed that HbA1c was negatively correlated with SUA in age stratification. However, we found that when HbA1c was about 12%, there was a positive correlation from a negative correlation. We found a significant negative correlation between HbA1c and SUA in men, but there were two U-shaped curves between HbA1c and SUA in women. A two-stage linear regression model determined the small inflection point to be 6.8%. For HbA1c 6.8%, every 1% increase in HbA1c was associated with a 9.86 *μ*mol/L decrease in SUA (95% CI: −13.25–−6.47). In the stratification of racial status, we found an inverted U-shaped relationship between HbA1c and SUA in all races. A two-stage linear regression model determined the inflection point.

**Figure 2:**
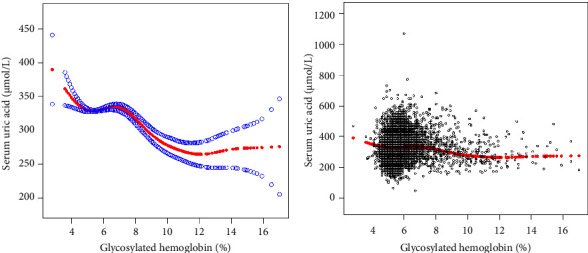
The association between Hb1Ac and SUA. (a) The solid red line represents the smooth curve fit between variables. Blue bands represent the 95% confidence interval from the fit. (b) Each black point represents a sample. Age, sex, race/ethnicity, education level, the ratio of family income to poverty, BMI, energy intake, protein intake, carbohydrate intake, dietary fiber intake, total fat intake, LDL-C, HDL-C, TG, TCHO, urine albumin, urine creatinine, urine albumin creatinine ratio, SBP, DBP, heavy alcohol consumption, smoking status, and minutes sedentary activity were adjusted.

**Figure 3:**
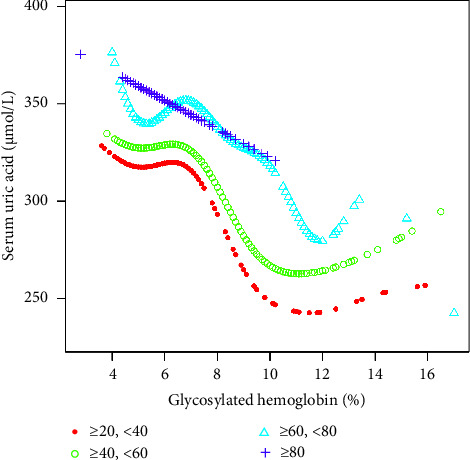
The association between HbA1c and SUA stratified by age. Sex, race/ethnicity, education level, the ratio of family income to poverty, BMI, energy intake, protein intake, carbohydrate intake, dietary fiber intake, total fat intake, LDL-C, HDL-C, TG, TCHO, urine albumin, urine creatinine, urine albumin creatinine ratio, SBP, DBP, heavy alcohol consumption, smoking status, and minutes sedentary activity were adjusted.

**Figure 4:**
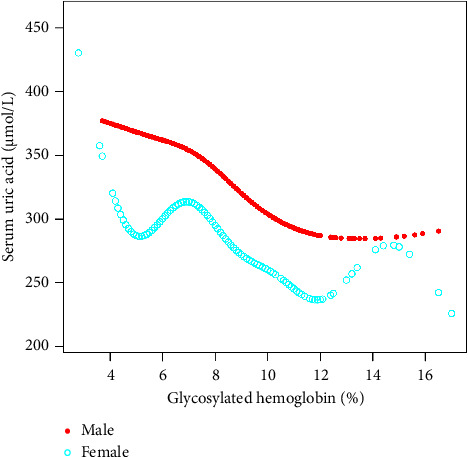
The association between HbA1c and SUA stratified by sex. Age, race/ethnicity, education level, the ratio of family income to poverty, BMI, energy intake, protein intake, carbohydrate intake, dietary fiber intake, total fat intake, LDL-C, HDL-C, TG, TCHO, urine albumin, urine creatinine, urine albumin creatinine ratio, SBP, DBP, heavy alcohol consumption, smoking status, and minutes sedentary activity were adjusted.

**Figure 5:**
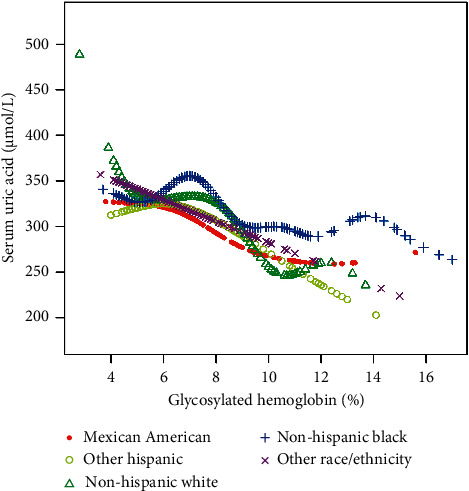
The association between HbA1c and SUA stratified by race/ethnicity. Age, sex, education level, the ratio of family income to poverty, BMI, energy intake, protein intake, carbohydrate intake, dietary fiber intake, total fat intake, LDL-C, HDL-C, TG, TCHO, urine albumin, urine creatinine, urine albumin creatinine ratio, SBP, DBP, heavy alcohol consumption, smoking status, and minutes sedentary activity were adjusted.

**Figure 6:**
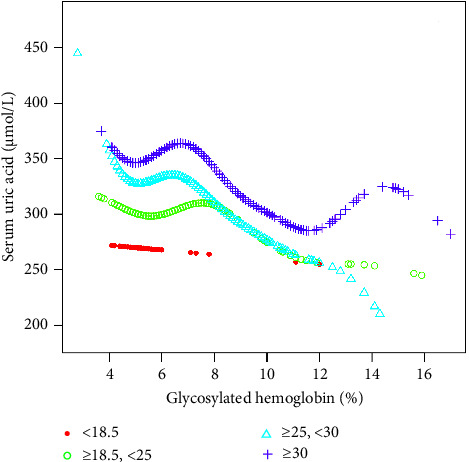
The association between HbA1c and SUA stratified by BMI. Age, sex, race/ethnicity, education level, the ratio of family income to poverty, energy intake, protein intake, carbohydrate intake, dietary fiber intake, total fat intake, LDL-C, HDL-C, TG, TCHO, urine albumin, urine creatinine, urine albumin creatinine ratio, SBP, DBP, heavy alcohol consumption, smoking status, and minutes sedentary activity were adjusted.

The inflection point appeared roughly at HbA1c, around 6.5%. In the age state stratification, we found an inverted U-shaped relationship between HbA1c and SUA in all age groups. We calculated the inflection point using a recursive algorithm and found that when age ≥20 and <40, the inflection point was 5.9%; When age ≥40 and <60, the inflection point was 6.2%; when age ≥60 and <80, the inflection point was 6.4%. When subgroup hierarchical analysis was carried out by race, we found a nonlinear relationship between HbA1c and SUA. By using the recursive algorithm to calculate the inflection point, when the race was Mexican American, the inflection point was 6.2%; when the race was other Hispanic, the inflection point was 6.1%; when the race was non-Hispanic White, the inflection point was 7.2%; and when the races were non-Hispanic Black and other race/ethnicity, the inflection point was 6.5%. At the same time, we found an inverted U-shaped relationship between HbA1c and SUA in BMI state stratification when BMI ≥ 18.5 kg/m^2^. A two-stage linear regression model determined the inflection point. We found that the inflection point roughly occurred at around 6.5% of HbA1c, similar to the results after stratification based on race and age. We speculated that the relationship between HbA1c and SUA was not influenced by age, race, or BMI. The threshold analysis results for gender and BMI subgroup stratification are shown in Tables 3–6. The results of smooth curve fitting and generalized additive model showed that there was a correlation between Hb1Ac and SUA risk. The details are shown in Figures 2–6.

## 4. Discussion

This was the first cross-sectional study to assess the American population's association between HbA1c and SUA. By adjusting other risk factors, results from the survey show a significant negative correlation between HbA1c and SUA in men, and there were multiple inflection points in women. When the HbA1c value was about 5%, the negative correlation between the two was changed to a positive correlation. When the HbA1c value was about 7%, the positive correlation between the two was converted to a negative correlation, and when the HbA1c value was about 12%, it was changed to a positive correlation.

HbA1c represents a reliable indicator of blood glucose levels in the body in the past 2-3 months. UA is the final product of human purine metabolism and has the characteristics of antioxidation and pro-oxidation [8]. The prooxidation and proinflammatory effects of UA affect the sensitivity of surrounding tissue cells to insulin, affect glucose homeostasis in the body, and promote the occurrence of diabetes [9, 10]. In previous studies, some epidemiological studies had shown that HbA1c was closely related to metabolic syndrome [11]. Several studies have confirmed that the association between SUA and HbA1c was inconsistent between men and women and between different blood glucose control levels [12–15]. Of course, the index of SUA often reflects gender specificity, which is more common in metabolic syndrome and acts on islet *β* cells [16, 17]. In the same level of hyperuricemia, the prevalence of hyperuricemia in men was generally higher than that in women [18]. Through the abovementioned studies, we believed there was a specific relationship between HbA1c and SUA, and the results differed in men and women. Studies have also shown that the main glycemic index and UA levels of men and women were inverted U-shaped, and the inflection point of men was earlier than that of women [6]. This study showed a significant negative correlation between HbA1c and SUA in adults over 20 years old in all three models of NHANES 2011–2020 data. At the same time, the relationship between HbA1c and SUA also showed different correlations in different genders, which had a specific correlation with previous studies.

The following pathophysiological mechanisms could explain the relationship between HbA1c and SUA. The body's intake of a large number of high-sugar, high-calorie, high-carbohydrate foods will not only lead to an increase in SUA levels but also easily lead to an increase in blood glucose. These reasons indirectly indicated that there must be a specific correlation between HbA1c level and SUA. Long-term high SUA levels in the body will affect endothelial function, stimulate the renin-angiotensin system [19], and produce an inflammatory response and oxidative stress response, thereby inhibiting insulin production, leading to glucose homeostasis imbalance and accelerating the occurrence and development of diabetes [1]. HbA1c was a product of the combination of glucose and hemoglobin, mainly involved in the occurrence and development of microvasculature in the body. Long-term hyperglycemia not only causes microvascular dysfunction but also increases nonenzymatic glycosylation, leading to changes in kidney structure and increased glomerular pressure, affecting glomerular filtration function, resulting in glomerulosclerosis, glomerular arteriosclerosis, renal interstitial fibrosis, and other outcomes. The kidney was the primary organ for excreting UA in the body. Abnormal kidney function had a particular impact on the excretion of UA. Therefore, damage to kidney function could reduce UA excretion, increasing SUA in the body. At the same time, when SUA levels increase, a large amount of urate salts accumulate in the renal interstitium and tubules, causing inflammation, damaging renal blood vessels, and affecting the filtration function of the glomeruli. Renal dysfunction could affect glucose and lipid metabolism, increasing blood sugar levels in the body.

Studies have also found that UA-related genes were closely related to insulin secretion in the Chinese population, indirectly affecting glucose metabolism in the body [20]. Decreased preglomerular resistance in diabetic patients helps to increase glomerular hyperfiltration, further promotes the excretion of UA, and leads to hypouricemia [21, 22]. Similarly, the islet function of diabetic patients was often damaged, which led to a decrease in insulin secretion in the body, downregulating the expression of renal urate transporters, reducing UA's reabsorption, and reducing SUA levels [23]. A cohort study in China found that higher SUA levels increased the risk of T2DM in average women [15]. At the same time, studies have also shown that SUA could interfere with the insulin signaling pathway at the receptor level through ENPP1 (hexanucleotide pyrophosphatase/phosphodiesterase 1) recruitment, affect glucose homeostasis, and change the level of HbA1c in the body, indicating that hyperuricemia will promote the occurrence of new diabetes [24]. We also found that the gender difference caused by the association between the two may also be related to gene mutations. The secondary allele of SLC2A9 had a more significant effect on reducing SUA in women, and the secondary allele of ABCG2 had a more substantial impact on increasing SUA in men [25]. Some researchers believed that the reason why there were gender differences in the two studies may be due to the urination of estrogen. Estrogen could upregulate the expression of mineralocorticoid receptors through glucocorticoid-dependent pathways and induce insulin resistance and hyperuricemia [26]. At the same time, May et al. studied that estrogen could also prevent diabetes by protecting islet *β* cells [27]. However, whether estrogen was involved in the gender-specific mechanisms between the two remains to be further clarified in the future.

Our research has some limitations. First of all, our research applied to the general population in the United States, and the conclusions were not necessarily applicable to other national populations, which limits the universality elsewhere. Second, this study was only a cross-sectional study, which belongs to the simultaneous determination of outcome and exposure factors, and cannot infer the causal relationship between HbA1c and SUA. Then, the data we collected could have been more comprehensive; we still need more extensive data research to explore this causal relationship further. Finally, although we adjusted the potential covariates, such as participants' dietary nutritional status, blood pressure, blood lipid levels, renal function status, smoking and drinking status, and physical activity levels, we cannot completely exclude the risk of bias caused by other confounding factors. Although there were some limitations, the results of this study were helpful to public health, and it was of particular significance to explore the correlation between HbA1c and SUA.

## 5. Conclusion

This study indicates that before about 7%, HbA1c in American adults was positively correlated with SUA and after about 7%, there was a negative correlation.

## Figures and Tables

**Table 1:** Baseline characteristics of all participants were stratified by quartiles of HbA1c.

Variables#	Total	Glycosylated hemoglobin (HbA1c)				*P* value
HbA1c quartile	5.8 ± 1.2	Q1	Q2	Q3	Q4	
		(2.8–5.2)	(5.2–5.5)	(5.5–5.9)	(5.9–17)	
Participants	7293	1306	1831	2149	2007	
Serum uric acid (*μ*mol/L)	328.3 ± 85.0	314.3 ± 83.9	316.6 ± 80.2	326.3 ± 79.9	350.1 ± 91.0	<0.001
Age (years)	49.3 ± 17.3	38.1 ± 15.0	43.1 ± 16.3	51.8 ± 16.5	59.4 ± 13.5	<0.001
Sex						<0.001
Male	3816 (52.3%)	635 (48.6%)	927 (50.6%)	1114 (51.8%)	1140 (56.8%)	
Female	3477 (47.7%)	671 (51.4%)	904 (49.4%)	1035 (48.2%)	867 (43.2%)	
Race (%)						<0.001
Mexican American	904 (12.4%)	147 (11.3%)	236 (12.9%)	263 (12.2%)	258 (12.9%)	
Other Hispanic	740 (10.2%)	110 (8.4%)	182 (9.9%)	225 (10.5%)	223 (11.1%)	
Non-Hispanic White	3087 (42.3%)	657 (50.3%)	854 (46.6%)	899 (41.8%)	677 (33.7%)	
Non-Hispanic Black	1604 (22.0%)	206 (15.8%)	277 (15.1%)	492 (22.9%)	629 (31.3%)	
Other race/ethnicity	958 (13.1%)	186 (14.2%)	282 (15.4%)	270 (12.6%)	220 (11.0%)	
Education level (%)						<0.001
Less than high school	1309 (18.0%)	143 (11.0%)	283 (15.5%)	403 (18.8%)	480 (23.9%)	
High school or GED	1651 (22.6%)	253 (19.8%)	371 (20.3%)	494 (23.0%)	533 (26.6%)	
Above high school	4333 (59.4%)	910 (69.7%)	1177 (64.3%)	1252 (58.3%)	994 (49.5%)	
The ratio of family income to poverty	2.6 ± 1.6	2.7 ± 1.7	2.7 ± 1.7	2.6 ± 1.6	2.5 ± 1.6	<0.001
BMI (kg/m^2^)	29.5 ± 7.2	26.9 ± 6.0	28.1 ± 6.5	29.5 ± 7.0	32.5 ± 7.8	<0.001
Energy intake (kcal)	2086.4 ± 842.9	2111.3 ± 863.1	2138.9 ± 868.2	2111.2 ± 850.4	1995.7 ± 790.0	<0.001
Protein intake (gm)	81.7 ± 36.5	82.6 ± 37.8	83.7 ± 39.3	82.0 ± 36.0	79.1 ± 33.4	<0.001
Carbohydrate intake (gm)	245.1 ± 107.3	247.4 ± 109.3	250.6 ± 109.3	249.2 ± 109.8	234.3 ± 100.6	<0.001
Dietary fiber intake (gm)	16.8 ± 9.5	16.7 ± 9.7	17.2 ± 10.3	17.0 ± 9.3	16.4 ± 8.7	0.253
Total fat intake (gm)	82.1 ± 39.2	81.0 ± 39.6	82.9 ± 39.8	83.1 ± 39.2	80.9 ± 38.3	0.152
LDL-C (mmol/L)	2.9 ± 0.9	2.7 ± 0.8	2.9 ± 0.9	3.0 ± 0.9	2.8 ± 1.0	<0.001
HDL-C (mmol/L)	1.4 ± 0.4	1.5 ± 0.5	1.5 ± 0.4	1.4 ± 0.4	1.3 ± 0.4	<0.001
TG (mmol/L)	1.3 ± 0.7	1.1 ± 0.7	1.1 ± 0.7	1.3 ± 0.7	1.5 ± 0.8	<0.001
TCHO (mmol/L)	4.8 ± 1.1	4.7 ± 1.0	4.9 ± 1.0	5.0 ± 1.1	4.8 ± 1.2	<0.001
Albumin, urine (mg/L)	46.7 ± 281.3	30.5 ± 225.0	19.9 ± 85.7	33.1 ± 232.6	96.4 ± 431.9	<0.001
Creatinine, urine (*μ*mol/L)	11575.3 ± 7113.9	11679.9 ± 7502.7	11567.5 ± 7357.4	11550.8 ± 7110.4	11540.5 ± 6619.2	0.711
Albumin creatinine ratio (mg/g)	42.7 ± 277.1	28.3 ± 188.5	17.2 ± 74.5	30.1 ± 247.9	89.0 ± 426.8	<0.001
SBP (mmHg)	123.4 ± 18.0	117.4 ± 15.5	118.8 ± 15.9	124.7 ± 18.2	129.9 ± 18.6	<0.001
DBP (mmHg)	71.0 ± 12.5	69.78 ± 11.4	70.66 ± 11.7	71.63 ± 12.1	71.50 ± 14.1	<0.001
Heavy alcohol consumption						0.008
Yes	1197 (16.4%)	198 (15.2%)	264 (14.4%)	374 (17.4%)	361 (18.0%)	
No	6096(83.6%)	1108 (84.8%)	1567 (85.6%)	1775 (82.6%)	1646 (82.0%)	
Smoking status						<0.001
Yes	3523 (48.3%)	537 (41.1%)	822 (44.9%)	1083 (50.4%)	1081 (53.9%)	
No	3770 (51.7%)	769 (58.9%)	1009 (55.1%)	1066 (49.6%)	926 (46.1%)	
Minutes sedentary activity (minute)	369.0 ± 202.0	381.4 ± 199.0	371.1 ± 202.1	354.6 ± 197.6	374.3 ± 207.6	<0.001

Mean ± SD for continuous variables: the *P* value was calculated by the weighted linear regression model. Percentage (%) for categorical variables: the *P* value was calculated by the weighted chi-square test. HbA1c, glycosylated hemoglobin; LDL-C, low-density lipoprotein cholesterol; HDL-C, high-density lipoprotein cholesterol; TG, triglyceride; TCHO, total cholesterol; SBP, systolic blood pressure; DBP, diastolic blood pressure.

**Table 2:** Association between Hb1Ac (%) and SUA (*μ*mol/L).

Exposure	Model 1	Model 2	Model 3
	*β* (95% CI) *P* value	*β* (95% CI) *P* value	*β* (95% CI) *P* value
Hb1Ac (%)	2.89 (1.19, 4.60) 0.0009	−0.82 (−2.44, 0.80) 0.3196	−7.93 (−9.49, −6.37) <0.0001
Glycosylated hemoglobin categories			
Q1 (2.8–5.2)	−29.20 (−49.64, −8.75) 0.0052	−16.77 (−34.34, 0.80) 0.0616	−14.82 (−31.13, 1.49) 0.0752
Q2 (5.2–5.5)	0.68 (−44.42, 45.79) 0.9763	−10.17 (−49.55, 29.21) 0.6128	−23.59 (−59.29, 12.11) 0.1954
Q3 (5.5–5.9)	25.63 (−5.31, 56.56) 0.1046	27.15 (−1.35, 55.66) 0.0620	3.38 (−23.20, 29.96) 0.8031
Q4 (5.9–17)	−9.87 (−12.33, −7.42) <0.0001	−9.42 (−11.79, −7.04) <0.0001	−11.79 (−14.08, −9.50) <0.0001
*P* for trend	<0.0001	<0.0001	<0.0001
Subgroup analysis stratified by sex			
Male	−3.07 (−5.17, −0.98) 0.0041	−3.75 (−5.94, −1.57) 0.0008	−10.38 (−12.51, −8.24) <0.0001
Female	7.43 (5.12, 9.74) <0.0001	2.76 (0.40, 5.13) 0.0219	−5.06 (−7.31, −2.81) <0.0001
Subgroup analysis stratified by age			
≥20, <40	3.54 (−0.55, 7.63) 0.0897	2.46 (−0.96, 5.88) 0.1581	−6.55 (−9.81, −3.29) <0.0001
≥40, <60	−1.55 (−4.11, 1.01) 0.2355	−3.03 (−5.37, −0.69) 0.0112	−9.44 (−11.68, −7.20) <0.0001
≥60, <80	2.26 (−0.93, 5.44) 0.1650	1.51 (−1.56, 4.58) 0.3349	−6.77 (−9.84, −3.71) <0.0001
≥80	2.84 (−7.80, 13.47) 0.6013	1.71 (−9.07, 12.49) 0.7565	−4.79 (−15.98, 6.40) 0.4018
Subgroup analysis stratified by race			
Mexican American	−2.23 (−6.34, 1.88) 0.2884	−5.63 (−9.45, −1.80) 0.0040	−10.58 (−14.24, −6.91) <0.0001
Other Hispanic	0.65 (−4.17, 5.47) 0.7912	−3.01 (−7.46, 1.44) 0.1850	−11.04 (−15.61, −6.47) <0.0001
Non-Hispanic White	7.19 (3.94, 10.43) <0.0001	2.05 (−1.06, 5.17) 0.1970	−9.38 (−12.39, −6.36) <0.0001
Non-Hispanic Black	2.99 (−0.13, 6.12) 0.0604	−0.41 (−3.30, 2.47) 0.7780	−5.95 (−8.70, −3.20) <0.0001
Other race/ethnicity	2.94 (−2.28, 8.16) 0.2705	0.54 (−4.43, 5.51) 0.8312	−6.67 (−11.64, −1.69) 0.0088

Model 1: no covariates were adjusted. Model 2: age, sex, and race were adjusted. Model 3: age, sex, race/ethnicity, education level, the ratio of family income to poverty, BMI, energy intake, protein intake, carbohydrate intake, dietary fiber intake, total fat intake, LDL-C, HDL-C, TG, TCHO, urine albumin, urine creatinine, albumin, creatinine ratio, SBP, DBP, heavy alcohol consumption, smoking status, and minutes sedentary activity were adjusted. In the subgroup analysis stratified by sex, age, and race/ethnicity, the model was not adjusted for sex, age, and race/ethnicity, respectively.

**Table 3:** Threshold effect analysis of HbA1c on SUA in males using the two-piecewise linear regression model.

Serum uric acid	Adjusted *β* (95% CI), *P* value
Male	
Model 1: fitting model by standard linear regression	−10.38 (−12.51, −8.24) <0.0001
Model 2: fitting model by two-piecewise linear regression	
Inflection point	6.4%
Glycosylated hemoglobin <6.4 (%)	0.27 (−5.72, 6.27) 0.9286
Glycosylated hemoglobin >6.4 (%)	−14.13 (−17.03, −11.22) <0.0001
*P* for the log-likelihood ratio test	<0.001

Age, race, education level, the ratio of family income to poverty, BMI, energy intake, protein intake, carbohydrate intake, dietary fiber intake, total fat intake, LDL-C, HDL-C, TG, TCHO, urine albumin, urine creatinine, albumin creatinine ratio, SBP, DBP, heavy alcohol consumption, smoking status, and minutes sedentary activity were adjusted.

**Table 4:** Threshold effect analysis of HbA1c on SUA in females using the two-piecewise linear regression model.

Serum uric acid	Adjusted *β* (95% CI), *P* value
Female	
Model 1: fitting model by standard linear regression	−5.06 (−7.31, −2.81) <0.0001
Model 2: fitting model by two-piecewise linear regression	
Inflection point	6.8%
Glycosylated hemoglobin <6.8 (%)	4.63 (−0.98, 10.25) 0.1057
Glycosylated hemoglobin >6.8 (%)	−9.86 (−13.25, −6.47) <0.0001
*P* for the log-likelihood ratio test	<0.001

Age, race, education level, the ratio of family income to poverty, BMI, energy intake, protein intake, carbohydrate intake, dietary fiber intake, total fat intake, LDL-C, HDL-C, TG, TCHO, urine albumin, urine creatinine, albumin creatinine ratio, SBP, DBP, heavy alcohol consumption, smoking status, and minutes sedentary activity were adjusted.

**Table 5:** Threshold effect analysis of HbA1c on SUA in BMI ≥25 kg/m^2^ and <30 kg/m^2^ using the two-piecewise linear regression model.

Serum uric acid	Adjusted *β* (95% CI), *P* value
BMI ≥25 kg/m^2^, <30 kg/m^2^	
Model 1: fitting model by standard linear regression	−10.45 (−13.49, −7.41) <0.0001
Model 2: fitting model by two-piecewise linear regression	
Inflection point	6.4%
Glycosylated hemoglobin <6.4 (%)	2.92 (−4.53, 10.37) 0.4419
Glycosylated hemoglobin >6.4 (%)	−16.41 (−20.70, −12.12) <0.0001
*P* for the log-likelihood ratio test	<0.001

Age, sex, race, education level, the ratio of family income to poverty, energy intake, protein intake, carbohydrate intake, dietary fiber intake, total fat intake, LDL-C, HDL-C, TG, TCHO, urine albumin, urine creatinine, albumin creatinine ratio, SBP, DBP, heavy alcohol consumption, smoking status, and minutes sedentary activity were adjusted.

**Table 6:** Threshold effect analysis of HbA1c on SUA in BMI ≥30 kg/m^2^ using the two-piecewise linear regression model.

Serum uric acid	Adjusted *β* (95% CI), *P* value
BMI ≥30 kg/m^2^	
Model 1: fitting model by standard linear regression	−7.02 (−9.28, −4.76) <0.0001
Model 2: fitting model by two-piecewise linear regression	
Inflection point	6.3%
Glycosylated hemoglobin <6.3 (%)	15.19 (7.45, 22.92) 0.0001
Glycosylated hemoglobin >6.3 (%)	−12.50 (−15.39, −9.60) <0.0001
*P* for the log-likelihood ratio test	<0.001

Age, sex, race, education level, the ratio of family income to poverty, energy intake, protein intake, carbohydrate intake, dietary fiber intake, total fat intake, LDL-C, HDL-C, TG, TCHO, urine albumin, urine creatinine, urine albumin creatinine ratio, SBP, DBP, heavy alcohol consumption, smoking status, and minutes sedentary activity were adjusted.

## Data Availability

Publicly available datasets were analyzed in this study. This data can be found here: https://www.cdc.gov/nchs/nhanes/. The independent variables, dependent variables, and covariates used in this study have the same data sources as previous related studies [28], but the conclusions are inconsistent due to the different specific data and research objectives used.
